# Pediatric Nephrology and Rheumatology Practice Patterns in Granulomatosis with Polyangiitis: A Midwest Pediatric Nephrology Consortium Study

**DOI:** 10.1155/2018/8413096

**Published:** 2018-08-05

**Authors:** Cristin D. W. Kaspar, Keia Sanderson, Seza Ozen, Priya S. Verghese, Megan Lo, Timothy E. Bunchman, Scott E. Wenderfer, Jason Kidd

**Affiliations:** ^1^Virginia Commonwealth University, Children's Hospital of Richmond, Pediatric Nephrology, Virginia, USA; ^2^University of North Carolina, Department of Medicine-Nephrology, North Carolina, USA; ^3^Hacettepe University, Department of Pediatrics, Turkey; ^4^University of Minnesota, Division of Pediatric Nephrology, Minnesota, USA; ^5^Baylor College of Medicine, Pediatrics-Renal, Texas, USA; ^6^Virginia Commonwealth University, Internal Medicine Nephrology, Virginia, USA

## Abstract

**Objective:**

To assess practice pattern similarities and differences amongst pediatric rheumatologists and nephrologists in the management of pediatric Granulomatosis with Polyangiitis (GPA).

**Methods:**

A voluntary survey was distributed to the Midwest Pediatric Nephrology Consortium Group (MWPNC) and an international pediatric rheumatology email listserv in 2016-2017. Data were collected on general practice characteristics and preferences for induction management under three clinical scenarios (A-C): newly diagnosed GPA with glomerulonephritis, GPA with rapidly progressive glomerulonephritis, and GPA with pulmonary hemorrhage. In addition, individual preferences for GPA maintenance medications, disease monitoring, and management of GPA with end-stage renal disease were ascertained.

**Results:**

There was a 68% response rate from the MWPNC membership and equal numbers of rheumatology respondents. Survey results revealed Rituximab plus Cyclophosphamide is a more common induction choice for rheumatologists than nephrologists in induction Scenarios A and B, whereas Cyclophosphamide is more commonly chosen by nephrologists in Scenario A. Plasmapheresis rates increased for Scenarios A, B, and C for both specialties, but were overall low. There was no clear consensus on the duration of maintenance therapy nor diagnostic work-up. Rheumatologists more frequently chose Rituximab for maintenance and induction compared to nephrologists. There was also a higher than expected proportion of Mycophenolate Mofetil use for both specialties.

**Conclusion:**

This survey has revealed important differences in the way that rheumatologists and nephrologists manage this disease. It highlights the need for well-designed clinical trials in pediatric GPA patients and reveals that both specialties must be represented during consensus-building and clinical trial design efforts.

## 1. Introduction

ANCA-associated vasculitis (AAV) is a disease which includes Granulomatosis with Polyangiitis (GPA) (formerly known as Wegener Granulomatosis), Microscopic Polyangiitis (MPA), and Eosinophilic Granulomatosis with Polyangiitis (EoGPA, formerly known as Churg Strauss Syndrome). Despite the commonality of ANCA association, the epidemiology, pathophysiology, course, and prognosis of these diseases are not uniform [1]. GPA is the most common AAV and while the incidence is low, it is rising from 0.2 to 1.2 per 100,000 [2], a trend that is consistent with our local experience.

The median age of GPA disease onset in childhood ranges from 11.7 to 14 years in the European Vasculitis registry and the international ARCHiVE cohort study, respectively [1, 3, 4]. In the ARCHiVE registry, 83% of newly diagnosed pediatric GPA patients presented with glomerulonephritis. The care of patients with GPA bridges the expertise of primarily two subspecialties, rheumatology and nephrology. It is presumed that current practice patterns of GPA amongst rheumatologists and nephrologists differ. To date there are no pediatric nephrology consensus guidelines supporting the management of the renal involvement of this disease. The European League Against Rheumatism (EULAR) released consensus statements in 2009 and updated in 2016 for AAV management, including recommendations for rapidly progressive glomerulonephritis (RPGN), pulmonary hemorrhage, and organ-threatening complications [5]. However these recommendations are not pediatric-specific and do not address management of end-stage renal disease in AAV, and it is unclear how familiar pediatric nephrologists are with the recommendations. Prompted by a recent increase in new pediatric GPA diagnoses in our local practice area and a lack of consensus guidance, we undertook a comparative survey to illustrate the practice patterns amongst pediatric nephrologists and rheumatologists for three different clinical presentations of pediatric GPA.

Pediatric GPA patients with renal involvement require a cooperative partnership between pediatric rheumatology and nephrology to care for the organ-threatening complications of this vasculitis and for the long-term management of immunosuppression. The goal of this survey is to identify commonalities in practice as well as to highlight disparate practice patterns that reveal specific education needs and areas that require further research.

## 2. Methods

This is an international descriptive survey of pediatric nephrologists and rheumatologists regarding the practice patterns of GPA. Members of the Midwest Pediatric Nephrology Consortium (MWPNC) and those of a pediatric rheumatology email listserv were asked to complete a voluntary survey on GPA practice patterns. The MWPNC is a collaborative clinical and translational research organization with 73 member centers represented across the United States, Puerto Rico, and Canada. The pediatric rheumatology listserv has an international email membership termed the “Pediatric Rheumatology Bulletin Board” hosted by McMaster University. A total of four email solicitations were sent between October 2016 and April 2017 to both memberships. Study objectives were presented to MWPNC members at a biannual research conference during this time period. Survey data were managed and collected using REDCap electronic data capture hosted at Virginia Commonwealth University (REDCap supported by Award Number UL1TR000058 from the NCRR). The full survey is available in Supplementary Materials 1 and included 5 sections.

Section 1 identified general characteristics such as the respondent's practice setting, the number of pediatric GPA patients currently followed, and whether their division had written consensus protocols for either the induction or maintenance treatment of GPA. In Section 2, respondents were asked to provide their first-line choice of induction medications depending on the presentation of a GPA patient in three different scenarios. They were first asked to consider management choices in a new GPA patient presenting with glomerulonephritis (Scenario A), a new patient with rapidly progressive glomerulonephritis (RPGN, Scenario B) as diagnosed using site-specific criteria, and a new patient with pulmonary hemorrhage (Scenario C). After induction differences were ascertained for Scenarios B and C, respondents were instructed to frame their remaining responses under Scenario A, for a presentation of a new diagnosis GPA with glomerulonephritis. In Sections 3 and 4, preferences were ascertained for maintenance Cyclophosphamide versus Rituximab, maintenance Azathioprine versus Mycophenolate Mofetil use, Trimethoprim-Sulfamethoxazole use, and clinical tools used for assessing initial and relapse disease activity. Finally, Section 5 was directed towards understanding the management of end-stage renal disease, including choice of dialysis modality, the timing of renal transplant once in remission, and the preferred choice of transplant immunosuppressive medications in GPA.

Multiple group proportions were compared by *χ*^2^ analysis, two sample proportions by Adjusted Wald test, with statistical significance level alpha <0.05. Statistical analysis was performed using JMP v.13.

## 3. Results

### 3.1. Survey Section 1: General Characteristics

Of the 145 responses to the survey, 7 were incomplete across all sections and excluded. The remaining 138 responses represented 42 unique rheumatology institutions and 3 unidentified, and 50 unique nephrology institutions and 2 unidentified which represented 68% of the MWPNC membership.  Table 1 lists practice setting differences and characteristics of the respondents. The primary management responsibility differed by specialty: 27% of the nephrology respondents (Neph) claimed they had primary management responsibility, whereas no rheumatology respondents (Rheum) reported that nephrology primarily managed the patients. Seven Rheum and 9 Neph respondents completed Section 1, but failed to complete Sections 2-5 and were therefore censored from the remainder of the analysis.

### 3.2. Survey Section 2: Induction Medications

Respondents were asked to select their individual preference for first-line induction medications under the three clinical scenarios (Scenarios A-C). One or multiple answers could be selected from the following choices: Cyclophosphamide (IV or PO), Rituximab, steroid (IV or PO), plasmapheresis, or other. After Scenario A choices were made, respondents were asked if their induction preferences would differ from Scenario A when considering Scenarios B or C, and if affirmed would prompt a follow-up set of questions with the same answer choices.  Table 2 summarizes the answer combinations and any significant specialty differences for each scenario.

Specialty-specific differences emerged for choice of induction agent across scenarios. Neph were more likely to choose Cyclophosphamide for Scenario A, whereas Rheum were more likely than Neph to use Rituximab together with Cyclophosphamide for both Scenarios A and B. Additionally there was an unique specialty difference for those who stated their induction agent would differ in Scenario C, a new diagnosis patient with pulmonary hemorrhage.

Experience level (categorized by <5 years including fellows in training, or ≥ 5 years) influenced Rheum responses for Cyclophosphamide and Rituximab in Scenario A only. Those with relatively more practice experience (≥5 years) chose Cyclophosphamide with more frequency (67.65% versus 23.81%, p=0.0008) and chose Rituximab with less frequency (78.18% versus 95.24%, p=0.0120). Plasmapheresis choice in Scenarios A-C and all other responses for Scenarios B and C were insignificant (p>0.05) when filtered by Rheum practice experience. There were no significant differences related to experience in Scenarios A-C for nephrologists. Next, induction responses were filtered for the number of GPA patients actively followed in the practice, categorized as 0-4 versus ≥ 5. There were no significant differences (p>0.05) for the induction agent chosen in either specialty when filtered by patient volume, except for Neph responses for Rituximab in Scenario C (pulmonary hemorrhage), where 64.29% of those with relatively higher patient volume chose Rituximab versus 35.71% with lower volume, p=0.02.

The remaining questions in Section 2 regarding dosing characteristics of the induction medications asked respondents to frame their answers in terms of Scenario A.

#### 3.2.1. Cyclophosphamide

The majority (65.3%) of respondents chose “>3 to 6 months” as the typical duration of induction Cyclophosphamide whereas 21.3% chose “>2 to 3 months.” The chosen interval was once per month in 78.5% whereas only 9.2% chose daily oral or every 2 weeks IV (specialty difference p>0.05). Both Rheum and Neph use an increasing dose protocol ending at 1000 mg/m^2^ (85%, p>0.05), but more Neph start at 500 mg/m^2^ (71.4%, specialty difference p=0.0358) whereas 36% of Rheum start at either 500 mg/m^2^ or 750 mg/m^2^. Conversely there was a consensus with no specialty difference for the starting dose, which was a median of 500 mg/m^2^, amongst those that chose both Cyclophosphamide and Rituximab for induction. Finally, 46.1% of respondents (specialty difference p>0.05) routinely refer adolescent patients to fertility specialists, whereas another 10.8% only refer if the patient nears a maximum cumulative dose limit of 10-15 g/m^2^.

#### 3.2.2. Rituximab

The method of dosing induction Rituximab differed significantly by specialty in terms of duration (p=0.0054), interval (p=0.0217), starting dose (p=0.0386), and set maximum dose (p=0.0033). The majority of Rheum (51.4%) dose at 750 mg/m^2^ every two weeks (35.1% dose at 375 mg/m^2^), whereas most Neph (77.8%) dose at 375 mg/m^2^ every four weeks. Rheum was more likely than Neph (84.6% versus 50%) to set a maximum dose of Rituximab of 1,000 mg, whereas 50% of Neph reported no set maximum dose.

#### 3.2.3. Steroids

The form of steroid chosen for induction differed by specialty (p=0.0068): “IV Methylprednisolone always” was chosen by 47.7% of Rheum and 75.9% by Neph, whereas “IV Methylprednisolone usually, depending on severity” was chosen by 38.6% Rheum and 22.2% Neph. Only 1.9% of Neph and 13.6% Rheum chose “PO steroid usually” and none “PO steroid always.” Three days of induction IV Methylprednisolone was the most common approach by both Rheum (89.5%) and Neph (88.5%). 92.2% overall dose IV Methylprednisolone using a mg/kg unit up to a set maximum amount of 1,000 mg (93.3%). Only 6 respondents (100% Neph) chose IV steroid as the lone induction agent for Scenario A, while none chose this as single therapy in Scenarios B or C.

#### 3.2.4. Plasmapheresis

While approximately 60% of respondents stated their induction choice would differ in a RPGN (Scenario B) or pulmonary hemorrhage (Scenario C) presentation versus Scenario A, there were similarities in the proportions of Rituximab and Cyclophosphamide selections with no difference by specialty. The difference arose in a higher selection of plasmapheresis: in Scenarios A, B, and C, respectively, the percentage of Rheum that chose plasmapheresis increased from 11.4%, 36.7%, and 51.9% versus Neph 21.4%, 42.9%, and 35.7%. Six and 11 respondents chose plasmapheresis as the lone induction agent for Scenarios B and C, respectively, which differed by specialty only for Scenario B.

### 3.3. Survey Section 3: Maintenance Medications

Respondents were next asked about duration of maintenance therapy, presuming patients remain free of relapse during that time. Most commonly respondents chose “>18 months to 2 years” (34.5%), followed by “>2 to 3 years” (26.7%) and “>12 to 18 months” (19.8%) with no difference by specialty (p=0.3335).

#### 3.3.1. Steroids

74.6% of respondents “always” use prednisone as a part of maintenance therapy; 22.8% use it “depending on severity of illness at presentation” (no specialty difference, p>0.05). The duration did differ by specialty (p=0.0022): 49.1% of Rheum wean prednisone “as quickly as possible once in remission, no set duration” versus 18% of Neph. The next most common response was “wean off by 6 months” (Rheum 29.1%, Neph 27.9%) and “wean off by 1 year” (Rheum 12.7%, Neph 23%). 18% of Neph versus 1.8% Rheum preferred to “remain on prednisone for the duration of maintenance therapy.”

#### 3.3.2. Rituximab

60% of Rheum versus 9.8% Neph chose Rituximab as first-line maintenance therapy (p<0.0001). Of note, 97% of Rheum and 60% Neph who chose Rituximab for first-line maintenance also chose Rituximab for first-line induction therapy (p=0.0403). The duration for maintenance Rituximab did not differ by specialty (p=0.3967): the most common response was “>6-12 months” (30.8%), followed by “>24 months” (23.1%) and “>18-24 months” (20.5%). Overall 61.5% of respondents dose Rituximab at intervals of once every 6 months, with some (25.6%) dosing it if the CD19 count rises above a set level (no specialty difference, p>0.05). Only 9 respondents described how they use this CD19 level in free text, and there was no consensus amongst the responses. 94.7% dose using mg/m^2^ unit. Most Neph begin dosing at 375 mg/m^2^ (83.3%), whereas Rheum start dosing at 750 mg/m^2^ (44.8%), p=0.0419. A consensus maximum set dose of 1,000 mg was common amongst both groups (82.1%).

#### 3.3.3. Azathioprine, Mycophenolate Mofetil, and Trimethoprim-Sulfamethoxazole

Overall 50.5% of respondents use Azathioprine over Mycophenolate Mofetil (MMF, 38.5%) for maintenance, and 11% overall chose “Other-not Azathioprine, MMF, or Rituximab primarily for maintenance.” While there was no difference by specialty for Azathioprine versus MMF choice, p=0.0654, Neph chose MMF in greater proportions than Rheum (45.8% versus 30%). Free text options were provided by 10 out of 12 respondents who chose “Other”: 3 reported using both Azathioprine and MMF simultaneously, 3 use Methotrexate, 3 stated they choose either Azathioprine or MMF situationally, and 1 misclassified and prefers Rituximab over Azathioprine/MMF. Of those that selected Rituximab for maintenance in the previous survey question (n=39), 47.1% also chose Azathioprine and 35.3% MMF with no difference by specialty (p>0.05).

The use of Trimethoprim-Sulfamethoxazole (TMP-SMX) for either* Pneumocystis jiroveci* (PJP) prophylaxis or as an additional component to maintenance therapy was next determined and did not differ by specialty (p>0.05). Overall, 81% of respondents use TMP-SMX in some way for GPA treatment: 37.1% of respondents use TMP-SMX purely for PJP prophylaxis, 37.1% use it for both PJP prophylaxis and to reduce relapse by decreasing sino-pulmonary carriage of staphylococcus or streptococcus bacteria, and 6.9% report using it to reduce the risk of relapse alone. The majority, 78%, do not monitor staphylococcus or streptococcus nasal carriage by nasal swab or culture, however, 10% check once at the start of treatment, and 12% test nasal swabs periodically. The TMP-SMX dosing interval did differ by specialty (p=0.0006), with Neph dosing it daily (48%, Rheum 11.4%), followed by three times a week (Neph 48%, Rheum 80%) and least common once per day on weekends only (Neph 4%, Rheum 9.1%).

### 3.4. Survey Section 4: Diagnostic Work-Up and Disease Monitoring

#### 3.4.1. Diagnostic Work-Up

88.3% of Neph “always” obtain a renal biopsy to confirm the diagnosis of GPA upon initial presentation, versus 47.3% of Rheum (p<0.0001). The extent of CT imaging to determine involvement of sino-pulmonary disease at the time of GPA diagnosis did not differ by specialty (p=0.0825). Most commonly a “LIMITED CT-scan depending on clinical presentation” (56.5%) was chosen, followed by “always obtain a FULL CT Head, Neck, Sinus, and Chest” (35.7%) or “no, never” (7.8%). Those that chose the limited CT-scan option were asked in follow-up whether they “always” obtained one or multiple specific organ systems: “head” (0%), “neck” (0%), “sinus” (Rheum 21%, Neph 79%, p=0.0266), “chest” (Rheum 37.1%, Neph 14.5%, p=0.0022), or “any combination of these but only if the patient has active symptoms” (24.6% overall, p=0.4934).

#### 3.4.2. Disease Monitoring

To define a GPA relapse, 41.2% overall use “either clinical characteristics or serological marker elevation,” 35.1% use “clinical characteristics and symptomatology only,” and 23.7% require “both clinical and serological marker elevation simultaneously” with no specialty difference, p>0.05. Specific diagnostic tools routinely employed included the following: ESR or CRP (Rheum 75.8%, Neph 50%, p=0.0019), ANCA titer (63.0%, p=0.1473), hematuria and/or proteinuria (73.9%, p=0.1038), and 9.4% (p=0.4970) use the Birmingham Vasculitis Activity Score. Three respondents wrote in use of the Pediatric Vasculitis Activity Score to assess relapse. The majority of respondents monitor ANCA titers “every 3 months” (64.4%), followed by “every 6 months” (13.9%), “only at times when a clinical relapse is suspected” (9.6%), “other” (8.7%), or “never” (3.5%) without specialty difference.

### 3.5. Survey Section 5: Management of GPA with End-Stage Renal Disease (ESRD)

The answer choices for the questions on dialysis and ESRD management included an option to select “unsure–I do not manage dialysis or transplant” (Rheum > Neph as expected, p<0.0001) and are reported in Table 3. It was expected that nearly all Rheum and some Neph would choose this ‘unsure' option as ESRD management would be outside the scope of practice for some. However, there was variability in the proportion selecting the ‘unsure' option for each question. Next, the ‘unsure' responses were excluded from analysis. Once a GPA patient is declared ESRD, the time in remission considered ‘safe' to receive a kidney transplant was “10-12 months” (34.7%), followed by “4-6 months” (25.0%) and “13-18 months” (23.6%) with no difference by specialty (p=0.2452). Although there was specialty difference in those that stated their transplant induction and maintenance choices would differ from non-GPA transplant recipients, proportions of the actual medications chosen were similar between groups. The majority (79.6%) chose anti-thymocyte globulin and IV steroid (81.5%) for induction, while majority for maintenance was MMF (98.2%), tacrolimus (94.5%), and PO steroid (67.3%).

## 4. Discussion

This survey revealed notably that, for GPA presenting with glomerulonephritis (Scenario A), an induction preference emerged for Rheum towards Rituximab + Cyclophosphamide and Neph towards Cyclophosphamide. The adult RAVE trial showed noninferiority of Rituximab to Cyclophosphamide for induction, as well as noninferiority in achieving complete remission rates at 6, 12, or 18 months in the follow-up RITUXVAS trial [6, 7]. However, the RAVE trial did not include dialysis-dependent patients at the time of presentation or those with serum creatinine >4 mg/dL, characteristics that might be seen in a presentation of RPGN as with Scenario B. No trial has compared combination therapy (Cyclophosphamide + Rituximab) versus monotherapy for induction to our knowledge. One possible reason for the preferred combination is perhaps a perception of a more severe disease in pediatric than adult patients with GPA and glomerulonephritis. An alternative explanation is that the combination choice may allow for a reduced dose of Cyclophosphamide to decrease toxicity, which is suggested by these data which showed more Rheum chose a starting dose of 750 mg/m^2^ for Cyclophosphamide alone but the median dose was 500 mg/m^2^ for the combination Cyclophosphamide + Rituximab.

The percentage of those choosing induction plasmapheresis for Scenarios B and C (roughly 35-50%) is not as high as would be expected if individuals follow or agree with the EULAR recommendations, where it is stated that plasma exchange should be considered for patients with RPGN or pulmonary hemorrhage (Statement #6, grades of evidence 1B and C, respectively) [5]. The rationale for choosing IV steroid as the lone induction agent for a minority of Neph respondents is unclear, but perhaps reflects a misunderstanding of the question or area for further investigation. Practice experience level and patient volume did differ for some induction responses, but did not have a consistent effect to explain differences revealed by this study.

There was little consensus overall on maintenance therapies or strategies; for example, no consensus emerged for the total duration of maintenance therapy, duration of steroid, or reason for using TMP-SMX. The overall 50% use of Azathioprine for maintenance was lower than expected, considering the findings of the IMPROVE [8] and WEGENT [9] trials that showed Azathioprine is superior to MMF or Methotrexate with fewer adverse events in adult GPA studies. Most Rheum use Rituximab for maintenance (and first-line induction) in line with the findings of the adult MAINRITSAN trial [10], where Rituximab was found to be superior for major relapses over 28 months compared to maintenance Azathioprine. Despite the majority of Rheum choosing Rituximab for both induction and maintenance, there was no consensus on the total duration of maintenance therapy revealing an area for investigation. The specialty differences in induction and maintenance preference found by this survey may reflect subspecialty favor of the medications based on experience with other diseases. For example, Cyclophosphamide and MMF are commonly used in systemic lupus erythematosus and other conditions and may explain the Neph preference for these agents. Additionally, if a provider is expecting that a GPA patient will eventually need renal transplantation, they might choose MMF for maintenance of GPA in planning the continuation of this medication for maintenance after transplantation.

Finally, Section 5 of the survey revealed some areas for consensus building but also areas that require further investigation. Limited adult data exist on renal transplant outcomes in GPA, and it is not clear whether the findings in the literature can be extrapolated to pediatrics. It is not unexpected that the rates of Rheum answering “unsure–I do not manage dialysis or transplant” were higher than Neph; however the absolute numbers choosing this option varied by question in this section. This may indicate that some rheumatologists do have practice preferences for some ESRD/dialysis management questions but not others, and is worth exploring in future studies. For example, 42 out of 55 Rheum were unsure of dialysis modality choice when considering infection risk, but only 32 out of 55 were unsure whether immunosuppression should be tapered sooner than non-dialysis GPA patients. The variance in Rheum selection of the unsure option may reflect the cooperative management relationship between Neph and Rheum for these patients, even after a patient has developed ESRD. Overall, most respondents do not wait for ANCA to be negative at time of transplant nor believe it influences risk of relapse which is supported by a retrospective adult study [11]. However 25% of respondents in this survey would transplant at less than 12 months in remission, which in the same study was associated with graft loss. ANCA disease relapse is reported to be low once patients are on chronic dialysis, but rates of infectious episodes while on chronic dialysis are high at 1.92/person-year [12, 13]. Some experts recommend hemodialysis over peritoneal dialysis for this reason, but no trials have specifically addressed this. Our survey did not find a preference for hemodialysis when considering infection risk. Lee et al. recommended from their analysis that continued immunosuppression after 4 months in ESRD dialysis-dependent patients is unlikely to be individually beneficial weighing the risks and benefits [14]. While a comprehensive study addressing the risk of infection versus benefit of continued immunosuppression while on dialysis has not been undertaken, the 75.6% response rate (excluding unsure responses) that immunosuppression course would not be shortened conflicts with the prior recommendations, revealing an area for future study. Co-management of these children even after ESRD is warranted based on the lack of data and variability of practice patterns.

## 5. Conclusion

This survey has revealed differences in practice patterns between specialties, with a few trends noted. Rituximab + Cyclophosphamide is a more common induction choice for rheumatologists than nephrologists in Scenarios A and B, whereas Cyclophosphamide is more commonly chosen by nephrologists in Scenario A. Plasmapheresis rates increased for Scenarios A, B, and C for both specialties, but were not as frequently chosen as one would expect if EULAR recommendations were followed. Variability found in this survey may be explained by a multitude of reasons: pediatric GPA patients with glomerulonephritis may be perceived to be more ill or not fit the recommendations extrapolated from adult trials; specialists may not be familiar with the most recent study results or recommendations as they encounter pediatric GPA patients rarely with majority following 2-4 in the practice; specialists may apply knowledge of management of other conditions to management of GPA contrary to recommendations; and the lack of evidence-based consensus statements or clinical trials for pediatric-specific GPA leads to varied practice patterns.

This survey has revealed important differences in the way that rheumatologists and nephrologists manage this disease. It has identified a need for improved dissemination of evidence-based results to influence practice patterns and reveals that both specialties must be represented during consensus-building and clinical trial design efforts. This study also underlines the need for well-designed controlled trials in pediatric GPA patients.

## Figures and Tables

**Table 1:** General characteristics of survey respondents.

	Total N	Rheum %	Neph %
Identified as a Rheumatologist / Nephrologist	138	44.9	55.1
Experience Level	138		
In practice >5 years		61.8	79.1
In practice <5 years		27.3	17.9
Fellows-in-training		10.9	3
Mid-Level Providers		0	0
Practice Setting	138		
University/Tertiary care/Academic		90.9	98.5
Community Hospital / Non-teaching / Non-academic		1.8	1.5
Private Practice		7.3	0
Supervise fellows directly	136	44.4	54.5
Institution has a Pediatric Nephrology Division	138	98.2	100
Institution has a Pediatric Rheumatology Division	135	96.3	89.2
Management			
Who manages pediatric GPA patients primarily at your institution?	138		
Primary Rheum		44 **∗**	9.5 **∗**
Primary Neph		0 **∗**	27 **∗**
Co-Management		56 **∗**	63.5 **∗**
Your institution does NOT have a written induction protocol	138	87.3	92.5
Your institution does NOT have a written maintenance protocol	138	90.9	97.0
Number of GPA patients currently followed in practice	138		
0		3.6	14.9
1		7.3	13.4
2-4		40.0	35.8
5-7		20.0	23.9
8-10		16.4	4.5
11+		12.7	7.5

*∗*Denotes significant difference by specialty p <0.0001.

**Table 2:** Characteristics of combination answer selections for Scenarios A-C.

	N	Overall % if no specialty difference	Rheum %	Neph %	**∗**p-value of specialty difference
**Scenario A**	**122**				
**Cyclophosphamide**	**77**	**∗**	**36.4**	**63.6**	**p=0.0114**
+ IV/PO Steroid	71	92.2			
+ Rituximab	26	*∗*	61.5	38.5	p=0.001
+ Plasmapheresis	14	19.5			
+ Rituximab + Plasmapheresis	9	11.7			
**Rituximab without Cyclophosphamide**	**39**	**60**			
+ IV/PO Steroid	36	92.3			
+ IV/PO Steroid + Plasmapheresis	6	15.4			
**IV/PO Steroid without Cyclophosphamide, Rituximab, or Plasmapheresis**	**6**	**∗**	**0**	**100**	**p<0.0001**

**Scenario B** My preferences would be different than that chosen in Scenario A	**75**	**61.5**			
**Cyclophosphamide**	**61**	**81.3**			
+ IV/PO Steroid	52	85.3			
+ Rituximab	23	**∗**	65.2	34.8	p=0.0185
+ Plasmapheresis	40	65.6			
+ Rituximab + Plasmapheresis	17	27.8			
**Rituximab without Cyclophosphamide**	**7**	**9.3**			
+ IV/PO Steroid	0	0			
+ IV/PO Steroid + Plasmapheresis	7	100			
**Plasmapheresis without Cyclophosphamide or Rituximab**	**6**	**∗**	**0**	**100**	**p<0.0001**
**Other** ^**i**^	**1**	**1.3**			

**Scenario C** My preferences would be different than that chosen in Scenario A	**72**	**∗**	**47.2**	**67.7**	**p=0.0232**
**Cyclophosphamide**	**45**	**63.4**			
+ IV/PO Steroid	40	88.9			
+ Rituximab	16	35.6			
+ Plasmapheresis	40	88.9			
+ Rituximab + Plasmapheresis	13	32.5			
**Rituximab without Cyclophosphamide**	**13**	**18.3**			
+ IV/PO Steroid	12	92.3			
+ IV/PO Steroid + Plasmapheresis	10	83.3			
**Plasmapheresis without ** **Cyclophosphamide or Rituximab**	**11**	**13.4**			
**Other** ^**ii**^	**3**	**4.1**			

^i^One subject chose ‘other' but did not describe treatment in free text.

^ii^One subject chose ‘other' but did not describe treatment in free text; two did not select any option.

**Table 3:** Management of GPA with end-stage renal disease.

Survey Question	Specialty, N	Yes, %	No, %	Unsure, %	p-value
Aside from routine considerations of modality, do you PREFER Hemodialysis over Peritoneal Dialysis (PD) because of the risk of infection from immunosuppression on PD?	Rheum, 55	9.1	14.5	76.4	P=0.019
	Neph, 63	17.4	74.6	7.94	

Once a patient is on dialysis, do you try to taper off the immunosuppression earlier than a non-dialysis GPA patient?	Rheum, 55	5.5	36.4	58.2	<0.0001
	Neph, 60	28.3	70.0	1.7	

Do you wait for ANCA titer to become negative before listing for transplant?	Rheum, 55	3.6	16.4	80.0	<0.0001
	Neph, 59	30.5	55.9	13.6	

Do you believe ANCA titer positivity AT THE TIME OF RENAL TRANSPLANT influences graft survival or vasculitis relapse rate?	Rheum, 55	7.3	9.1	83.6	<0.0001
	Neph, 58	25.8	51.7	22.4	

Is your renal transplant INDUCTION immunosuppression for a patient with GPA any different than a non-GPA renal transplant patient?	Rheum, 54	3.7	3.7	92.6	<0.0001
	Neph, 59	18.6	64.4	17.0	

Is your renal transplant MAINTENANCE immunosuppression for a patient with GPA any different than a non-GPA renal transplant patient?	Rheum, 55	3.6	3.6	92.7	<0.0001
	Neph, 59	20.3	66.1	13.6	

## Data Availability

The data used to support the findings of this study are available from the corresponding author upon request.

## Abbreviations

MWPNC: Midwest Pediatric Nephrology Consortium
GPA: Granulomatosis with polyangiitis
AAV: ANCA-associated vasculitis
MMF: Mycophenolate Mofetil
TMP-SMX: Trimethoprim-sulfamethoxazole.

## Supplementary Materials

Supplementary Materials Practice pattern REDCap survey form.
