# Supplementary material for: Pediatric Nephrology and Rheumatology Practice Patterns in Granulomatosis with Polyangiitis: A Midwest Pediatric Nephrology Consortium Study
Source: Int J Nephrol. 2018 Aug 5;2018:8413096. doi: 10.1155/2018/8413096 (PMC6098870; doi:10.1155/2018/8413096)
Supplement: Supplementary Materials — Practice pattern REDCap survey form. [file 8413096.f1.pdf]

Granulomatosis with Polyangiitis (GPA) Practice Patterns amongst Pediatric Nephrologists and Rheumatologists

VCU IRB Study ID: HM20007791. Virginia Commonwealth University, Children's Hospital of Richmond, Pediatric Nephrology

Study Objective: To study the variability in practice patterns amongst pediatric rheumatologists and nephrologists in treating pediatric granulomatosis with polyangiitis (GPA, aka Wegener's Granulomatosis).

Principal Investigator: Dr. Cristin Kaspar, cristin.kaspar@vcuhealth.org, Ph 804-628-3866, CHoR at VCU Pediatric Nephrology

Co-P.I.: Dr. Keia Sanderson, UNC Pediatric Nephrology

Dr. Timothy E. Bunchman, timothy.bunchman@vcuhealth.org, CHoR at VCU Pediatric Nephrology

Dr. Megan Lo, megan.lo@vcuhealth.org, CHoR at VCU Pediatric Nephrology

Dr. Sarah Hoffman, sarah.hoffman@vcuhealth.org, CHoR at VCU Pediatric Rheumatology

This is a voluntary survey that will query your first-line choices in: induction, maintenance, relapse definitions, duration of therapy, and other practice patterns in this disease.

Total participation time estimated to be 10 minutes.

By clicking on this link, you agree to participate in the study. No patient-specific or participant-specific data will be requested or collected, and all responses will remain anonymous.

Your participation in this survey is greatly appreciated! Please email cristin.kaspar@vcuhealth.org for any questions regarding the survey.

SECTION 1 of 5: GENERAL CHARACTERISTICS

The following section pertains to general characteristics of your institution / practice.

***Granulomatosis Polyangiitis will hereafter be abbreviated "GPA"**

What is the name of your institution / practice group? ______________________________

Note: this information will only be used to track percent response. Name of institution will NOT be included in any publication materials and will only be viewed by the PI of the study.

Are you a pediatric:
- ◯ rheumatologist
- ◯ nephrologist

Does your institution have a pediatric rheumatology division?
- ◯ Yes
- ◯ No

How many providers are currently employed in the pediatric rheumatology division? ______________________________

** If there is no rheumatology division, enter "0"

Does your institution have a pediatric nephrology division?
- ◯ Yes
- ◯ No

How many providers are currently employed in the pediatric nephrology division? ______________________________

** If there is no nephrology division, enter "0"

Are you a:
- ◯ MD/DO practicing >5 years
- ◯ MD/DO practicing < 5 years
- ◯ MD/DO fellow in training
- ◯ Mid-level provider NP/PA

What type of institution do you practice in?
- ◯ University / Tertiary Care Center (teaching, academic)
- ◯ Community Hospital (non-teaching, non-academic)
- ◯ Private practice (majority outpatient)

Do you supervise fellows directly in your division?
- ◯ Yes
- ◯ No

Who manages pediatric GPA patients primarily at your institution?
- ◯ Pediatric Nephrology
- ◯ Pediatric Rheumatology
- ◯ co-managed, or combined Pediatric Nephrology/Rheumatology clinic
- ◯ Adult Nephrology
- ◯ Adult Rheumatology
- ◯ co-managed or combined Adult Nephrology/Rheumatology clinic
- ◯ Other

(Choose 'Other' if you would like to expand on your answer)

Expand on your answer 'Other': __________________________________

How many pediatric GPA patients are currently followed by your practice?

- ◯ 0
- ◯ 1
- ◯ 2-4
- ◯ 5-7
- ◯ 8-10
- ◯ 11+

Does your division have a WRITTEN consensus protocol for INDUCTION medications for treatment of GPA?

- ◯ Yes
- ◯ No

Does your division have a WRITTEN consensus protocol for MAINTENANCE medications for treatment of GPA?

- ◯ Yes
- ◯ No

SECTION 2 of 5: INDUCTION MEDICATIONS

The following questions address your INDIVIDUAL first-line preference in Induction medications for a NEW DIAGNOSIS GPA WITH GLOMERULONEPHRITIS.

**** There are 2 separate questions specifically about Induction with a presentation of: Rapidly Progressive Glomerulonephritis (RPGN) and one of Pulmonary Hemorrhage.**
After these questions please return to framing your responses for a presentation of new diagnosis GPA with glomerulonephritis.

NEW DIAGNOSIS GPA WITH GLOMERULONEPHRITIS

What medication(s) do you prefer to use as first-line for INDUCTION?

You may select multiple answers

☐ IV or PO cyclophosphamide
☐ rituximab
☐ IV or PO steroid
☐ plasmapheresis
☐ other

Expand on your answer 'Other':

____

If a new diagnosis GPA with RAPIDLY PROGRESSIVE GLOMERULONEPHRITIS (RPGN) requires INDUCTION, would your treatment be any different?

◯ Yes
◯ No

GPA WITH RPGN

What medication(s) do you prefer to use as first-line for INDUCTION?

You may select multiple answers

☐ IV or PO cyclophosphamide
☐ rituximab
☐ IV or PO steroid
☐ plasmapheresis
☐ other

Explain your answer 'Other'

____

Please explain the difference - specifically, dosing and duration of the 'additional' induction medications, and rationale of your choice.

____

If a new diagnosis GPA with PULMONARY HEMORRHAGE requires INDUCTION, would your treatment be any different?

◯ Yes
◯ No

GPA WITH PULMONARY HEMORRHAGE

What medication(s) do you prefer to use as first-line for INDUCTION?

You may select multiple answers

☐ IV or PO cyclophosphamide
☐ rituximab
☐ IV or PO steroid
☐ plasmapheresis
☐ other

Explain your answer 'Other'

____

Please explain the difference - specifically, dosing and duration of the 'additional' induction medications, and rationale of your choice.

____

NEW DIAGNOSIS GPA WITH NEPHRITIS

What form of Cyclophosphamide do you prefer to use for INDUCTION?

☐ IV
☐ PO

What is the typical duration of INDUCTION Cyclophosphamide?

○ < 1 month
○ 1 month
○ >1 to 2 months
○ >2 to 3 months
○ > 3 to 6 months
○ > 6 to 12 months
○ > 12 to 18 months
○ > 18 to 24 months

At what interval do you prescribe the INDUCTION Cyclophosphamide?

○ Daily (oral)
○ Once per month (IV)
○ Every 2 weeks (IV)
○ Other

Expand on your answer 'Other'

____

Do you use an increasing-dose protocol for Cyclophosphamide?

[e.g. start at 500mg/m2, then 750 mg/m2, stop at 1000 mg/m2]

○ Yes
○ No

What unit of measure do you base Cyclophosphamide dosing on?

○ mg/m2
○ mg/kg

What DOSE do you prescribe for Cyclophosphamide INDUCTION (based on the above unit of measure)?

Note: If you use an increasing-dose protocol, answer with the STARTING dose.

[Answer in # form, e.g. "600"]

____

What is the FINAL dose for Cyclophosphamide Induction in the increasing-dose protocol?

[Answer in # form, e.g. "600"]

____

Do you routinely refer ADOLESCENT patients to fertility specialists (sperm banking or oocyte cryopreservation) when on Cyclophosphamide treament?

○ Yes
○ No
○ Only if nearing the maximum recommended cumulative dosing limit of 10-15 g/m2.

What is the typical DURATION for INDUCTION Rituximab?

[Note: There will be separate questions for Maintenance Rituximab duration]

○ 2 weeks
○ 4 weeks
○ 2 to 3 months
○ > 3 to 6 months
○ > 6 to 12 months

What is the INTERVAL you use for INDUCTION Rituximab?

- ◯ once weekly
- ◯ once every 2 weeks
- ◯ once every 4 weeks
- ◯ once every 6 months
- ◯ Other

Explain your answer 'Other' for Rituximab Induction Interval

____

What is the unit of measure you use to dose INDUCTION Rituximab?

- ◯ mg/m2 up to a set maximum daily amount
- ◯ mg/kg up to a set maximum daily amount

What is the Dose you use for INDUCTION Rituximab (based on the above unit of measure)?

[Answer in # form, e.g. "375"]

____

What is the set maximum daily amount you use to dose INDUCTION Rituximab?

- ◯ 500 mg
- ◯ 1,000 mg
- ◯ 2,000 mg
- ◯ No set amount

What form, and how do you dose STEROIDS for INDUCTION?

- ◯ IV Methylprednisolone always
- ◯ IV Methylprednisolone usually, but depends on severity of illness at presentation
- ◯ PO Prednisone/prednisolone always
- ◯ PO Prednisone/prednisolone usually, but depends on severity of illness at presentation

How many days of IV methylprednisolone do you usually prescribe for induction?

- ◯ 1-2 days
- ◯ 3 days
- ◯ 5 days
- ◯ Other (type-in)

Expand on your answer 'Other':

____

What is the unit of measure you use to dose IV Methylprednisolone for INDUCTION?

- ◯ mg/kg up to a set maximum daily amount
- ◯ mg/m2 up to a set maximum daily amount

What is the dose of IV Methylprednisolone that you prescribe (based on the unit of measure above)?

[Answer in # form, e.g. "2"]

____

What is the set maximum daily amount you use for IV Methylprednisolone for INDUCTION?

- ◯ up to 500 mg
- ◯ up to 1,000 mg
- ◯ up to 2,000 mg
- ◯ no set amount

What is the DURATION for PO prednisone/prednisolone when used for INDUCTION

[Note: There will be separate questions for Maintenance PO prednisone]

- ◯ 3 days or less
- ◯ 4-5 days
- ◯ 6-7 days
- ◯ 8-30 days
- ◯ 1-3 months

What is the INTERVAL you use for PO prednisone/prednisolone when used for INDUCTION

- ◯ Daily
- ◯ Every other day

What is the unit of measure you use for PO prednisone/prednisolone when used for INDUCTION?

- ◯ mg/kg up to a set max daily amount
- ◯ mg/m2 up to a set max daily amount

What is the DOSE you use for PO prednisone/prednisolone when used for INDUCTION (based on the unit specified above)

[Note: Answer in # form, e.g. "2"]

____

What is the maximum daily dose you use for PO prednisone/prednisolone when used for INDUCTION (based on the unit specified above)

[Note: Answer in # form, e.g. "2"]

- ◯ 60 mg
- ◯ 80 mg
- ◯ 100 mg
- ◯ No set daily dose

SECTION 3 of 5: MAINTENANCE MEDICATIONS

The following questions address your first-line choice in maintenance medication, and pertain to your INDIVIDUAL preference and practice.

Do you use PO prednisone/prednisolone for maintenance?

[Note: Taper schedule addressed in later questions]

- ◯ Always
- ◯ Depends on severity of illness at presentation
- ◯ Other (type-in)

Expand on your answer 'Other': ____

As the patient's clinical status allows, in what time frame do you AIM to taper OFF prednisone?

- ◯ As quickly as possible once in remission, no set duration
- ◯ By 3 months
- ◯ By 6 months
- ◯ By 1 year
- ◯ Remains on prednisone for the duration of maintenance therapy
- ◯ Other (type-in)

Expand on your answer 'Other': ____

Do you use Rituximab for first-line MAINTENANCE?

- ◯ Yes
- ◯ No

What is the DURATION for MAINTENANCE rituximab?

- ◯ 0 to 6 months
- ◯ > 6 months to 12 months
- ◯ > 12 months to 18 months
- ◯ > 18 months to 24 months
- ◯ > 24 months

What is the INTERVAL you use to dose MAINTENANCE Rituximab?

- ◯ once per month
- ◯ once every 6 months
- ◯ once per year
- ◯ depends on CD19 level, I dose it if CD19 rises above a set level
- ◯ other

What is the set level for CD19 count, on which you base the decision to re-dose Rituximab? ________________________________

Explain your answer choice 'Other' for Rituximab dosing interval ____

What is the unit of measure you use to dose MAINTENANCE Rituximab?

- ◯ mg/m2
- ◯ mg/kg

What is the dose you prescribe for MAINTENANCE Rituximab (based on the unit of measure above)? ________________________________

[Note: Answer in # form, e.g. "375"]

What is the maximum set dose you use for MAINTENANCE Rituximab?

- ◯ 500 mg
- ◯ 1,000 mg
- ◯ 2,000 mg
- ◯ no set maximum dose

Do you use Rituximab for first-line INDUCTION also?

- ◯ Yes
- ◯ No

What do you use for first-line induction if your first-line maintenance preference is Rituximab?

You may select more than 1 response if you use multiple induction agents at once

- ☐ IV cyclophosphamide
- ☐ PO cyclophosphamide
- ☐ IV methylprednisolone
- ☐ PO prednisone/prednisolone
- ☐ IV rituximab
- ☐ Other

Explain your answer 'Other'

____

Do you use AZATHIOPRINE or MYCOPHENOLATE MOFETIL for first-line maintenance?

- ◯ azathioprine
- ◯ mycophenolate mofetil
- ◯ other (type-in) - select this if you do not use rituximab, azathioprine, or mycophenolate mofetil primarily for maintenance

Expand on your answer 'Other':

____

Do you use Trimethoprim-Sulfamethoxazole (Bactrim) as part of the maintenance therapy?

- ◯ No
- ◯ Yes, but purely for infection / PJP prophylaxis
- ◯ Yes, to decrease the risk of relapse by decreasing sino-pulmonary carriage of staph/strep bacteria
- ◯ Yes, used for BOTH PJP prophylaxis AND to reduce relapses

Do you monitor Staph or Strep carriage in nasal passages through swab/culture?

- ◯ No
- ◯ Yes once at the start of treatment
- ◯ Yes, I test periodically

Do you use the swab/culture result to determine whether to treat with Bactrim as part of the maintenance regimen?

- ◯ No, I will use Bactrim for maintenance regardless of result.
- ◯ Yes, I will only use Bactrim for maintenance if there is positive staph/strep carriage

At what interval do you prescribe Bactrim (trimethoprim/sulfamethoxazole)?

- ◯ once per day on weekends only
- ◯ once daily (7 days a week)
- ◯ three times per week

How long do you AIM to keep patients on maintenance medication, assuming they remain in remission during that time period without complications, before you try weaning off maintenance?

- ◯ 1 to 6 months
- ◯ > 6 to 12 months
- ◯ > 12 to 18 months
- ◯ > 18 months to 2 years
- ◯ > 2 years to 3 years
- ◯ indefinitely, I keep them on maintenance unless they develop a complication
- ◯ Other (type-in)

Expand on your answer 'Other':

____

SECTION 4 of 5: Assessing GPA disease activity and defining relapse

Do you always obtain a renal biopsy to confirm a diagnosis of GPA?

- ◯ Yes
- ◯ No

Do you always obtain CT-scan to assess for extent of sino-pulmonary disease once a diagnosis of GPA is made?

- ◯ Yes I always obtain a FULL CT Head, Neck, Sinus, and Chest/Lungs
- ◯ Yes but I obtain a LIMITED CT-scan depending on clinical presentation [This answer choice prompts a follow-up question]
- ◯ No, never

If you obtain a LIMITED set of CT-scans at time of diagnosis, what do you obtain?

[Note: you may select multiple answers]

- ☐ CT Head always
- ☐ CT Neck always
- ☐ CT Sinus always
- ☐ CT Chest/Lungs always
- ☐ Any combination of the above, but ONLY if the patient has active symptoms.

How do you define a GPA relapse: i.e. do you think there are pure serologic, pure clinical relapses, or must a relapse be a mixture of both?

- ◯ Clinical characteristics and symptomatology
- ◯ Serological marker elevation
- ◯ EITHER clinical OR serological marker elevation
- ◯ BOTH clinical AND serological marker elevation are required simultaneously to declare relapse

Which of the following do you routinely assess to diagnose a GPA relapse?

[Note: you may select more than 1 answer]

- ☐ Clinical symptomatology (informal assessment), e.g. presence of fatigue, new rash, sinopulmonary symptoms.
- ☐ Elevation in Birmingham Vasculitis Activity Score (BVAS)
- ☐ Elevation of serum ESR or CRP
- ☐ Elevation of ANCA titer
- ☐ Hematuria and/or proteinuria
- ☐ Other

Explain how you use the BVAS score to define relapse

____

Expand on your answer 'Other':

____

At what time interval do you typically monitor ANCA titers?

- ◯ Never
- ◯ Every 3 months
- ◯ Every 6 months
- ◯ Once per year
- ◯ Only at times I am concerned about a clinical relapse
- ◯ Other (type-in)

Expand on your answer 'Other':

____

SECTION 5 of 5: Management of End-Stage Renal Disease in GPA

When considering dialysis modality for a pediatric patient with GPA:
Aside from routine considerations of modality, do you PREFER Hemodialysis over Peritoneal Dialysis (PD) because of the risk of infection from immunosuppression on PD?

- ◯ Yes
- ◯ No Preference
- ◯ Unsure - I don't manage dialysis

Once a patient is on dialysis, do you try to taper off the immunosuppression earlier than a non-dialysis GPA patient?

- ◯ Yes
- ◯ No
- ◯ Unsure - I don't manage dialysis

If you have a pediatric patient with GPA and end stage renal disease, how much time in remission do you feel is 'safe' to list for Status 1/Active Kidney Transplant?

- ◯ If in remission < 1 month, list for status 1 transplant immediately
- ◯ 1-3 months
- ◯ 4-6 months
- ◯ 7-9 months
- ◯ 10-12 months
- ◯ 13-18 months
- ◯ 19-24 months
- ◯ Other (type-in)
- ◯ Unsure - I don't manage transplant

Expand on your answer 'Other':

____

Do you wait for ANCA titer to become negative before listing for transplant?

- ◯ Yes
- ◯ No
- ◯ Unsure - I don't manage transplant

Do you believe ANCA titer positivity AT THE TIME OF RENAL TRANSPLANT influences graft survival or vasculitis relapse rate?

- ◯ Yes
- ◯ No
- ◯ Unsure - I don't manage transplant

Is your Renal Transplant INDUCTION immunosuppression for a patient with GPA any different than a non-GPA renal transplant patient?

- ◯ Yes
- ◯ No
- ◯ Unsure - I don't manage transplant

What is your Renal Transplant INDUCTION immunosuppression regimen of choice for someone with GPA?

[Note: you may select more than 1 answer if you use multiple of these agents for induction at once]

- ☐ anti-thymocyte globulin or other Polyclonal agents
- ☐ basiliximab or other Monoclonal agents
- ☐ IV steroid
- ☐ mycophenolate mofetil
- ☐ Other
- ☐ Unsure - I don't manage transplant

Expand on your answer 'Other':

____

Is your Renal Transplant MAINTENANCE immunosuppression for a patient with GPA any different than a non-GPA renal transplant patient?

- ◯ Yes
- ◯ No
- ◯ Unsure - I don't manage transplant

What is your Renal Transplant MAINTENANCE immunosuppression regimen of choice for someone with GPA?

- ☐ mycophenolate mofetil
- ☐ tacrolimus or sirolimus
- ☐ prednisone
- ☐ cyclosporine A
- ☐ azathioprine
- ☐ Other
- ☐ Unsure - I don't manage transplant

Expand on your answer 'Other':

____
